# Validation of the Prognostic Stage of American Joint Committee on Cancer Eighth Edition Staging Manual in Invasive Lobular Carcinoma Compared to Invasive Ductal Carcinoma and Proposal of a Novel Score System

**DOI:** 10.3389/fonc.2020.01471

**Published:** 2020-08-18

**Authors:** Shuning Ding, Yu Zong, Caijin Lin, Lisa Andriani, Weilin Chen, Deyue Liu, Weiguo Chen, Yafen Li, Kunwei Shen, Jiayi Wu, Li Zhu

**Affiliations:** ^1^Department of General Surgery, Comprehensive Breast Health Center, Ruijin Hospital, Shanghai Jiao Tong University School of Medicine, Shanghai, China; ^2^Stanford Cancer Institute, Stanford University School of Medicine, Stanford, CA, United States

**Keywords:** tumor staging, pathological prognostic stage, invasive lobular carcinoma, score system, predictive performance

## Abstract

**Purpose:** The objective of this study was to evaluate the American Joint Committee on Cancer (AJCC) pathological prognostic stage among patients with invasive ductal carcinoma (IDC) and invasive lobular carcinoma (ILC) and to propose a modified score system if necessary.

**Methods:** Women diagnosed with IDC and ILC during 2010–2015 in the Surveillance, Epidemiology, and End Results (SEER) database were retrospectively identified. Disease-specific survival (DSS) and overall survival (OS) were estimated by Kaplan–Meier method. Predictive performances of different staging systems were evaluated based on Harrell concordance index (C-index) and Akaike Information Criterion (AIC). Multivariate Cox models were conducted to build preferable score systems.

**Results:** A total of 184,541 female patients were included in the final analyses, with a median follow-up of 30.0 months. In IDC cohort, the pathological prognostic stage (C-index, 0.8281; AIC, 110274.5) was superior to the anatomic stage (C-index, 0.8125; AIC, 112537.0; *P* < 0.001 for C-index) in risk stratification with respect to DSS. In ILC cohort, the prognostic stage (C-index, 0.8281; AIC, 7124.423) didn't outperform the anatomic stage (C-index, 0.8324; AIC, 7144.818; *P* = 0.748 for C-index) with respect to DSS. Similar results were observed with respect to OS. The score system defined by anatomic stage plus grade plus estrogen receptor and progesterone receptor (AS+GEP) allows for better staging (C-index, 0.8085; AIC, 7178.448) for ILC patients.

**Conclusion:** Compared with anatomic stage, the pathological prognostic stage provided more accurate stratification for patients with IDC, but not for patients with ILC. The AS+GEP score system may fit ILC tumors better.

## Introduction

Tumor staging is of critical significance in risk stratification and prognosis prediction for breast cancer. Since its first publication in 1977, the American Joint Committee on Cancer (AJCC) Cancer Staging Manual has been periodically revised and updated to improve its predictive accuracy in stratifying patients outcomes (1). Historically, the standardized classification system was solely based on anatomic extent of primary breast tumor, lymph node, and metastasis (TNM) (1). With better understanding toward tumor biology, the importance of adding biological factors as complementary to conventional staging system has been recognized (2–5). Therefore, AJCC 8th edition staging manual introduced the prognostic stage system (PS) by incorporating biomarkers including estrogen receptor (ER) and progesterone receptor (PR) expression, human epidermal growth factor receptor 2 (HER2) status, tumor grade, as well as multigene assays when available, with TNM classification, while maintaining the TNM-based anatomic stage system(AS) (6). And after further analysis based on National Cancer Database (NCDB), the AJCC Breast Expert Panel provided an updated version of the breast staging manual to further refine the patient stratification (7). The PS has been previously validated in invasive breast cancer and proved to be superior to the AS (8–11). However, the prognostic value of the PS in different histology subtypes of breast cancer has not been evaluated yet, which requires further validation.

Invasive ductal carcinoma (IDC) and invasive lobular carcinoma (ILC) are the two most common histological types of invasive breast cancer, with IDC occurring in about three fourths of patients and ILC approximately accounting for 10–12% of all cases (12–15). As reported in previous studies, the clinical and biological characteristics were different between IDC and ILC. Compared to patients with IDC, patients with ILC are generally associated with an older age at diagnosis, larger tumor size, lower tumor grade, and more frequent lymph node involvement (12, 14, 16). Regarding biomarkers, ILC is more likely to be ER/PR-positive and HER2-negative (12, 14). From the treatment perspective, ILC was reported to be less sensitive to chemotherapy than IDC (13, 17), even in the genomic intermediate/high risk group (18). Despite these distinctive differences, studies specially focused on ILC were relatively insufficient. Until now, the prognostic value of PS has not been exclusively evaluated in this specific histological type of breast cancer.

In our study, the objective is to assess and compare the predictive performances of AS and PS in both IDC cohort and ILC cohort, and furthermore, to propose a modified prognostic staging score system in case the current PS did not perform ideally in the ILC subtype.

## Patients and Methods

### Data Source and Study Cohort

This retrospective study was conducted using data from the Surveillance, Epidemiology, and End Results (SEER) database which collected data from 18 population-based cancer registries, approximately representing 28% of the US population.

Patients meeting the following inclusion criteria were identified as potentially eligible patients: (1) female; (2) years at diagnosis from 2010 to 2015; (3) histologically confirmed breast cancer as the primary and only malignant tumor; (4) histological breast cancer subtypes were IDC (8500/3) and ILC (8520/3) according to International Classification of Diseases for Oncology, Third Edition (ICD-O-3); (5) having received a mastectomy or a breast-conserving surgery (BCS) as surgical treatments. Patients without available information on biomarkers including tumor grade, ER, PR, and HER2 status, and patients diagnosed by death certificate or autopsy only were further excluded.

Data retrieved from SEER database included as follows: age at diagnosis, race, histological subtypes, anatomic features including tumor size and lymph node involvement, biomarkers including tumor grade, ER, PR, and HER2 status and treatment information such as surgical procedure, chemotherapy, and radiation. Patients were assigned to different stages according to the AS and PS in the AJCC 8th edition staging manual (7). AS was defined by traditional TNM classification, while PS was defined by TNM classification and additional biomarkers including tumor grade, ER, PR, and HER2 status. The PS was divided into clinical prognostic stage and pathological prognostic stage in the updated version of staging manual. In this study, pathological prognostic stage was applied and PS referred to pathological prognostic stage.

### Statistical Analysis

The demographic and tumor characteristics were compared between patients with IDC and ILC using Pearson's Chi-square test or Fisher's exact test when necessary. The disease-specific survival (DSS) was calculated from the time of diagnosis to the time of death from breast cancer. The overall survival (OS) was calculated from the time of diagnosis to the time of death from any causes. The survival was estimated by Kaplan–Meier method and were compared by log-rank test. The Cox proportional hazards model was utilized to analyze the univariate and multivariate association of each potential prognostic factor with DSS and OS, and to calculate hazard ratio (HR) and 95% confidence interval (CI). The predictive performances of different staging systems were quantified and compared based on Harrell's concordance index (C-index) and Akaike information criterion (AIC), which were calculated from Cox models adjusted by age at diagnosis, race, surgery types, receipt of chemotherapy, and radiation. A higher C-index indicates a better discriminatory ability among each staging system (19). A lower AIC indicates a more effective model in predicting outcomes (20).

A two-tailed *P* < 0.05 was considered statistically significant. All of the statistical analyses were conducted using STATA (version 14.0, College Station, TX, US).

### Model Building

Corresponding with other published studies (3, 5), DSS was determined as the clinical endpoint when the staging score systems were created. AS was considered as a reference stage to derive the novel scoring system. Univariate analyses were conducted to evaluate the association between DSS and potential prognostic factors including tumor grade, ER status, PR status, and HER2 status. The Cox models based on AS were performed to assess the prognostic significance of adding other candidate factors. Only factors associated with DSS in univariate analyses (*p* < 0.05) could be included in multivariate Cox models. Therefore, the first model was based on AS. The second model incorporated AS and tumor grade. The third model included AS, tumor grade, ER, and PR status. HER2 status was excluded because it was not significantly associated with DSS, which would be further explained in the results section. Scoring systems were created according to the multivariate analysis results of the three Cox models. Scores were assigned to each independent prognostic factor of DSS (*p* < 0.05). For binary variables, the comparison group with significant impact on DSS was assigned one point. For ordinal variables, the comparison groups determined to have a significant impact on DSS with an HR between 1.01 and 4 were assigned one point, variables with an HR between 4.01 and 8 were assigned two points, variables with an HR between 8.01 and 12 were assigned three points and variables with an HR over 12 were assigned four points. The final score was obtained by summing scores for all independent predictors of DSS. Finally, three score system were created. The first score system was solely based on AS. The second score system included the AS and tumor grade (AS+G). The third score system evolved the AS, tumor grade, ER status, and PR status (AS+GEP). The predictive performances were quantified and compared using C-index and AIC (19, 20).

## Results

### Clinical and Biological Features

A total of 201,075 patients in SEER database met the eligible criteria. Among 180,652 patients diagnosed with IDC, 10,413 (5.7%) patients with unknown ER, PR, or HER2 status and 4,155 (2.3%) patients without tumor grade information were further excluded. Among 20,423 patients diagnosed with ILC, 970 (4.7%) patients with unknown ER, PR or HER2 status and 996 (4.9%) patients without tumor grade information were further excluded. A total of 184,541 female patients were included in the final study. The IDC cohort consisted of 166,084 (89.9%) patients while the ILC cohort consisted of 18,457 (10.1%) patients. The median age of the whole population was 60 years (range 18–98). The demographic, clinicopathological characteristics, and treatment disposition of each cohort were summarized in Table 1.

**Table 1:** Demographic, clinicopathological characteristics, and treatment disposition of IDC cohort and ILC cohort.

**Characteristics**	***N*** **(%)**		***P*-value**
	**IDC (*N* = 166,084)**	**ILC (*N* = 18,457)**	
Age at diagnoses			<0.001
≤ 60	88,550 (53.3)	7,854 (42.6)	
>60	77,534 (46.7)	10,603 (57.4)	
Race			<0.001
White	130,750 (78.7)	15,640 (84.7)	
Black	18,421 (11.1)	1,617 (8.8)	
Others^a^	16,913 (10.2)	1,200 (6.5)	
Surgery			<0.001
BCS	101,113 (60.9)	9,309 (50.4)	
Mastectomy	647,971 (39.1)	9,148 (49.6)	
pT			<0.001
T1	104,800 (63.1)	9,268 (50.2)	
T2	50,343 (30.3)	6,450 (34.9)	
T3	7,488 (4.5)	2,493 (13.5)	
T4	3,453 (2.1)	246 (1.3)	
pN			<0.001
N0	114,835 (69.1)	12,381(67.1)	
N1	38,660 (23.3)	4,003 (21.7)	
N2	8,470(5.1)	1,157 (6.3)	
N3	4,119 (2.5)	916 (5.0)	
Grade			<0.001
1	35,599 (21.4)	5,383 (29.2)	
2	68,992 (41.5)	11,565 (62.7)	
3	61,493 (37.0)	1,507 (8.2)	
ER status			<0.001
Positive	134,603 (81.0)	18,154 (98.4)	
Negative	31,481 (19.0)	303 (1.6)	
PR status			<0.001
Positive	118,418 (71.3)	15,646 (84.8)	
Negative	47,666 (28.7)	2,811 (15.2)	
HER2 status			<0.001
Negative	138,122 (83.2)	17,612 (95.4)	
Positive	27,962 (16.8)	845 (4.6)	
Molecular subtype			<0.001
HR+HER2-	117,012 (70.5)	17,400 (94.3)	
HR-HER2+	8,416 (5.1)	69 (0.4)	
HR+HER2+	19,546 (11.8)	776 (4.2)	
TNBC	21,110 (12.7)	212 (1.1)	
Radiation			<0.001
No	66,454 (40.0)	7,707 (41.8)	
Yes	99,630 (6 0.0)	10,750 (58.2)	
Chemotherapy			<0.001
No/unknown	92,350 (55.6)	12,552 (68.0)	
Yes	73,734 (44.4)	5,905 (32.0)	

*BCS, breast conserving surgery; ER, estrogen receptor; IDC, invasive ductal carcinoma; ILC, invasive lobular carcinoma; HER2, human epidermal growth factor receptor 2; HR, hormone receptor; PR, progesterone receptor; pT, pathological T; pN, pathological N; TNBC, triple negative breast cancer*.

a *Including American Indian, Alaskan Native, Asian and Pacific Islander*.

Distinct differences in clinicopathological features between IDC cohort and ILC cohort were observed. There was a significant higher percentage of patients aged 60 and younger in IDC cohort than ILC cohort (53.3 vs. 42.6%, *P* < 0.001). Patients with ILC were more likely to have mastectomy than BCS compared to those with IDC (49.6 vs. 39.1%, *P* < 0.001). Significant differences were observed in pT stage and pN stage distribution among patients with IDC and ILC (*P* < 0.001). Patients with ILC was associated with larger tumor size and more lymph node involvement. With regard to biomarkers, ER-positive tumors, and PR-positive tumors were more common in ILC cohort than in IDC cohort (ER: 98.4 vs. 81.0%, *P* < 0.001; PR: 84.8 vs. 71.3%, *P* < 0.001). Conversely, HER2-positive tumors (IDC vs. ILC: 16.8 vs. 4.6%, *P* < 0.001) and grade 3 tumors (IDC vs. ILC: 37.0 vs. 8.2%, *P* < 0.001) were more common in IDC cohort than in ILC cohort.

### Stage Distribution and Migration

Patients in both cohorts were restaged according to the AS and the PS proposed in the AJCC 8th edition staging manual. The distribution of stages applying the AS and the PS were listed in Table 2. By using the AS, percentage of patients in stage IA, IB, IIA, IIB, IIIA, IIIB, IIIC was 51.7, 2.7, 23.0, 11.9, 6.6, 1.7, and 2.5% in IDC cohort, while the percentage was 42.1, 1.8, 24.9, 14.2, 10.9, 1.1, and 5.0% in ILC cohort. By using the PS, percentage of patients in stage IA, IB, IIA, IIB, IIIA, IIIB, IIIC was 61.7, 15.5, 10.4, 4.0, 4.3, 2.1, and 2.1% in IDC cohort, while the percentage was 65.9, 19.3, 4.1, 2.9, 5.7, 1.7, and 0.5% in ILC cohort.

**Table 2:** Distribution and survival outcomes by anatomic stages and prognostic stages in IDC (*N* = 166,084) and ILC cohort (*N* = 18,475).

**Stage**	**IDC cohort**			**ILC cohort**		
	***N* (%)**	**4-years DSS**	**4-years OS**	***N* (%)**	**4-years DSS**	**4-years OS**
**AS**						
IA	85,807 (51.7)	98.71	95.68	7,779 (42.1)	99.23	95.99
IB	4,500 (2.7)	97.74	95.63	326 (1.8)	98.63	94.57
IIA	38,186 (23.0)	95.25	91.31	4,596 (24.9)	97.43	92.75
IIB	19,762 (11.9)	91.23	87.28	2,628 (14.2)	96.34	92.13
IIIA	10,879 (6.6)	84.53	81.46	2,010 (10.9)	92.05	87.14
IIIB	2,831 (1.7)	75.33	68.83	202 (1.1)	74.65	68.15
IIIC	4,119 (2.5)	71.82	67.22	916 (5.0)	77.55	73.94
**PS**						
IA	102,448 (61.7)	98.86	95.70	12,156 (65.9)	98.80	95.27
IB	25,722 (15.5)	95.72	92.20	3,553 (19.3)	95.49	91.22
IIA	17,329 (10.4)	91.00	87.13	757 (4.1)	95.11	89.06
IIB	6,599 (4.0)	86.71	83.40	541 (2.9)	91.68	86.15
IIIA	7,092 (4.3)	82.30	78.28	1,049 (5.7)	83.01	77.15
IIIB	3,431 (2.1)	76.00	71.12	313 (1.7)	72.33	69.12
IIIC	3,463 (2.1)	57.43	52.38	87 (0.5)	52.53	48.09

*AS, anatomic staging system; DSS, disease-specific survival; IDC, invasive ductal carcinoma; ILC, invasive lobular carcinoma; OS, overall survival; PS, prognostic staging system*.

Overall, after applying the PS, there were 40.5% patients who underwent stage changing: 13,215 (7.2%) patients were upstaged and 61,462 (33.3%) patients were downstaged. Compared to the IDC cohort, it was less likely for patients with ILC to have upstaging (IDC vs. ILC: 7.9 vs. 0.6%), yet more common to have downstaging (IDC vs. ILC: 31.4 vs. 50.5%). Detailed information about the stage migration when switching from the AS to the PS was exhibited in Table 3.

**Table 3:** Migration of patients from anatomic stage to prognostic stages (top, IDC cohort; bottom, ILC cohort).

**Stage**		**AJCC 8th PS**						
		**IA**	**IB**	**IIA**	**IIB**	**IIIA**	**IIIB**	**IIIC**
AJCC 8th AS	IA	78,102 (91.02)	7,705 (8.98)	0	0	0	0	0
	IB	4,217 (93.17)	283 (6.29)	0	0	0	0	0
	IIA	18,740 (49.08)	6,334 (16.59)	13,112 (34.34)	0	0	0	0
	IIB	1,389 (7.03)	7,578 (38.35)	3,435 (17.38)	4,540 (22.97)	2,820 (14.27)	0	0
	IIIA	0	3,822 (35.12)	782 (7.19)	2,059 (18.93)	2,467 (22.68)	211 (1.94)	1,538 (14.14)
	IIIB	0	0	0	0	760 (26.85)	1,242 (43.87)	829 (29.28)
	IIIC	0	0	0	0	1,045 (25.37)	1,978 (48.02)	109 6(26.61)
**Stage**		**AJCC 8th Prognostic stage**						
		**IA**	**IB**	**IIA**	**IIB**	**IIIA**	**IIIB**	**IIIC**
AJCC 8th AS	IA	7,724 (99.29)	55 (0.71)	0	0	0	0	0
	IB	325 (99.69)	1 (0.31)	0	0	0	0	0
	IIA	3,569 (77.65)	500 (10.88)	527 (11.47)	0	0	0	0
	IIB	538 (20.47)	1,507 (57.34)	208 (7.91)	367 (13.96)	8 (0.30)	0	0
	IIIA	0	1,491 (74.18)	22 (1.09)	174 (8.66)	296 (14.73)	16 (0.80)	11 (0.55)
	IIIB	0	0	0	0	127 (62.87)	53 (26.42)	22 (10.89)
	IIIC	0	0	0	0	618 (67.47)	244 (26.64)	54 (5.90)

*AJCC, American Joint Committee on Cancer; AS, anatomic stage; PS, prognostic stage*.

*Percent frequency in the boxes represents the distribution of prognostic stages in the same anatomic stage (for e.g., among patients with IDC, 91.02% of anatomic stage IA patients remained stage IA and 8.98% of them were upstaged to stage IB when applying prognostic stage system)*.

*Red boxes represent patients having an upstaged prognostic stage, and green boxes represent those who were downstaged after applying prognostic stage system, whereas those in blue maintained an unchanged stage*.

### Survival Outcomes and Comparison of Predictive Performance

**Figure 1:**
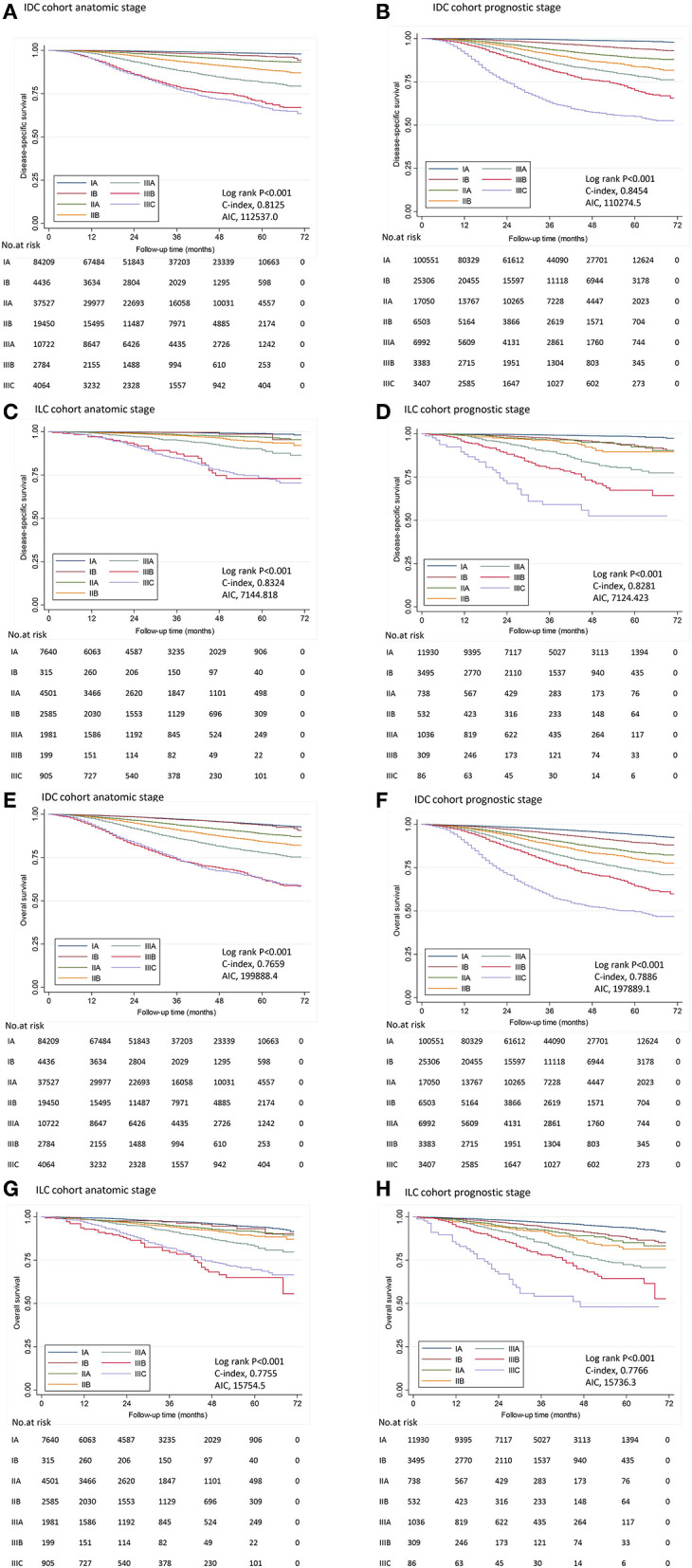
Disease-specific survival and overall survival by stage in IDC and ILC cohort. Kaplan–Meier survival plots demonstrated DSS **(A–D)** and OS **(E–H)** in different stages determined by the 8th AJCC staging manual in IDC cohort and ILC cohort. IDC, invasive ductal carcinoma; ILC, invasive lobular carcinoma; AIC, Akaike information criterion.

In this study, the median follow-up duration was 30.0 months (range, 0–71 months). The estimated 4-years DSS and OS according to different stages and histological subtypes were summarized in Table 2 and the corresponding survival curves were demonstrated in Figure 1. According to the univariate analyses, the DSS of different stage groups by the AS and the PS were significantly different in both cohorts (all *P* < 0.001, Table 2). Similar results were observed for OS. Cox proportional hazard regression models adjusted with age, race, surgery types, and receipt of chemotherapy and radiation was performed for subsequent statistical analyses. According to multivariate analyses using stage IA as reference, the differences in DSS and OS among stage groups remained significant in both cohorts (all *P* < 0.001, Table 4).

**Table 4:** Hazard Ratios for disease-specific survival and overall survival by stages in IDC cohort and ILC cohort.

		**IDC**				**ILC**			
		**Anatomic stage**		**Prognostic stage**		**Anatomic stage**		**Prognostic stage**	
**Endpoint**	**Stage**	**HR (95% CI)**	***P***	**HR (95%CI)**	***P***	**HR (95% CI)**	***P***	**HR (95%CI)**	***P***
DSS	IA	1		1		1		1	
	IB	1.98 (1.55–2.51)	<0.001	4.20 (3.77–4.67)	<0.001	2.21 (0.68–7.17)	<0.001	5.01 (3.70–6.78)	<0.001
	IIA	3.61 (3.27–3.98)	<0.001	8.79 (7.93–9.74)	<0.001	3.60 (2.39–5.42)	<0.001	4.67 (2.94–7.44)	<0.001
	IIB	6.92 (6.24–7.67)	<0.001	13.81 (12.26–15.55)	<0.001	6.88 (4.51–10.50)	<0.001	7.26 (4.62–11.42)	<0.001
	IIIA	13.58 (12.21–15.10)	<0.001	20.20 (18.12–22.52)	<0.001	17.85 (11.80–27.01)	<0.001	19.34 (13.98–26.78)	<0.001
	IIIB	23.19 (20.43–26.33)	<0.001	28.73 (25.48–32.40)	<0.001	45.10 (26.75–76.06)	<0.001	32.08 (22.32–46.12)	<0.001
	IIIC	27.38 (24.47–30.63)	<0.001	62.94 (56.52–70.09)	<0.001	50.23 (33.03–75.78)	<0.001	72.55 (46.12–114.17)	<0.001
OS	IA	1		1		1		1	
	IB	1.22 (1.03–1.45)	<0.001	2.31 (2.16–2.47)	<0.001	1.74 (0.97–3.11)	<0.001	2.69 (2.24–3.23)	<0.001
	IIA	2.31 (2.18–2.46)	<0.001	4.08 (3.81–4.36)	<0.001	1.86 (1.53–2.78)	<0.001	2.58 (1.92–3.45)	<0.001
	IIB	3.85 (3.60–4.12)	<0.001	5.80 (5.31–6.34)	<0.001	2.89 (2.30–3.62)	<0.001	3.66 (2.70–4.95)	<0.001
	IIIA	6.96 (6.46–7.49)	<0.001	8.58 (5.31–6.34)	<0.001	6.35 (5.04–7.99)	<0.001	8.09 (6.51–10.05)	<0.001
	IIIB	11.73 (10.68–12.88)	<0.001	11.80 (10.78–12.91)	<0.001	13.57 (9.55–19.25)	<0.001	10.16 (7.68–13.43)	<0.001
	IIIC	13.20 (12.16–14.32)	<0.001	25.75 (23.84–27.80)	<0.001	12.78 (10.04–16.26)	<0.001	25.25 (17.46–36.51)	<0.001

*CI, confidence interval; HR, hazard ratio; IDC, invasive ductal carcinoma; ILC, invasive lobular carcinoma; DSS, disease-specific survival; OS, overall survival*.

In the IDC cohort, C-index was 0.8454 for the PS, vs. 0.8125 for the AS; and AIC was 110274.5 for the PS, vs. 112537.0 for the AS according to the Cox model using DSS as endpoint (Figure 1). The PS represented a significant higher C-index (*P* < 0.001) and a lower AIC compared to AS, which indicated that the PS was a more effective model in predicting prognosis among patients with IDC. Similar results were seen for OS (Figure 1).

In the ILC cohort, C-index was 0.8281 for the PS, vs. 0.8324 for the AS according to the Cox model using DSS as endpoint. There was no significant difference in C-index between the AS and the PS (*P* = 0.748). Moreover, AIC was similar between the AS and the PS (7144.8 vs. 7124.4), indicating PS was not superior to AS in predicting prognosis. Similar results were seen for OS (Figure 1).

Likewise, the results of statistical assessment in the subgroup patients after excluding those received chemotherapy and among patients with ER-positive and HER2-negative tumors also manifested that PS provided better risk stratification with significantly higher C-index and lower AIC compared to AS in IDC cohort but not in ILC cohort (Supplement Tables 1, 2).

### New Score System for ILC

Revised score systems were applied in ILC tumors to optimize risk stratification among patients with ILC.

The results of univariate and multivariate analyses for potential prognostic factors associated with DSS were summarized in Table 3. According to univariate analyses, HER2 status was not related to DSS (*P* = 0.253), while the AS, tumor grade, ER status, and PR status were all prognostic factor for DSS in ILC cohort (all *P* < 0.001). Therefore, multivariate models including these prognostic factors were constructed to assess the prognostic value of each factor and determine score assignment. The first Cox model was based on AS. The second Cox model combined AS and tumor grade. The third Cox model incorporated AS, tumor grade, ER, and PR status. According to multivariate analyses, stage IIA, IIB, IIIA, IIIB, and IIIC patients had worse DSS in comparison with stage IA patients. Tumor grade, ER status, and PR status were all independently associated with DSS in each model (all *P* < 0.001). Accordingly, three score systems were established and scores were assigned for these independent predictors based on hazard ratio as described in the method. The detailed information about point assignments for independent predictors of DSS were shown in Table 5.

**Table 5:** Univariate and multivariate analyses for factors associated with DSS and point assignment in ILC cohort.

**Factor**	**Univariate analysis**		**Multivariate analysis**				**Point**
			**Model AS+G**		**Model AS+GEP**		
	**HR**	**P**	**HR**	**P**	**HR**	**P**	
**Stage**							
IA	1	/	1		1	/	0
IB	1.84	<0.001	1.75	0.354	1.87	0.269	0
IIA	3.34	<0.001	3.15	<0.001	3.15	<0.001	1
IIB	5.31	<0.001	4.91	<0.001	5.04	<0.001	2
IIIA	10.74	<0.001	9.76	<0.001	9.82	<0.001	3
IIIB	30.16	<0.001	26.49	<0.001	23.86	<0.001	4
IIIC	31.41	<0.001	27.90	<0.001	26.06	<0.001	4
**Grade**							
1	1	/	1		1	/	0
2	1.75	<0.001	1.43	<0.001	2.38	0.014	1
3	4.30	<0.001	2.47	<0.001	2.06	<0.001	1
**ER Status**							
Positive	1	/			1	/	0
Negative	6.85	<0.001			2.51	<0.001	1
**PR Status**							
Positive	1	/			1	/	0
Negative	2.47	<0.001			1.75	<0.001	1
**HER2 Status**							
Negative	1	/					
Positive	1.27	0.253					

*AS+G, anatomic stage plus grade; AS+GEP, anatomic stage plus grade plus estrogen receptor plus progesterone receptor; DSS, disease-specific survival; ER, estrogen receptor; HER2, human epidermal growth factor receptor 2; HR, hazard ratio; ILC, invasive lobular carcinoma; PR, progesterone receptor*.

**Figure 2:**
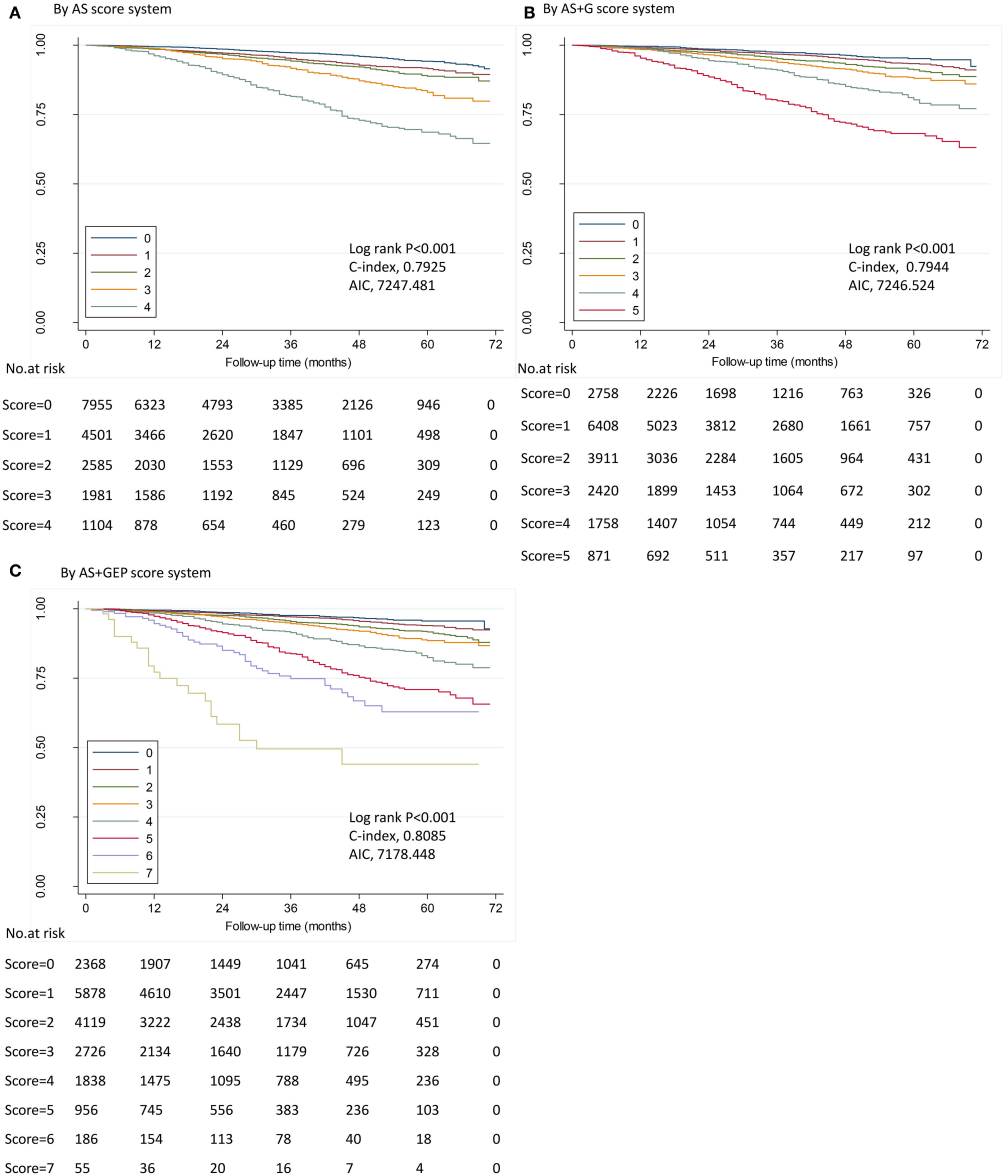
Disease-specific survival by score in ILC cohort. Kaplan–Meier survival plots demonstrated DSS in different stages determined by five score systems in ILC cohort. ILC, invasive lobular carcinoma; AIC, Akaike information criterion. **(A)** by anatomic stage (AS) score system; **(B)** by AS plus tumor grade (AS+G) score system; **(C)** by AS plus tumor grade plus estrogen receptor (ER) status plus progesterone receptor (PR) status (AS+GEP) score system. IDC, invasive ductal carcinoma; ILC, invasive lobular carcinoma; AIC, Akaike information criterion.

The first score system was solely based on AS. The second score system included the AS and tumor grade (AS+G). The third score system evolved the AS, tumor grade, ER status, and PR status (AS+GEP). Figure 2 demonstrated the DSS curves for each score system. By using all three score systems, there were significant differences in DSS among patients in different score groups (all *P* < 0.001). The AS+GEP score system exhibited the higher C-index (0.8085 vs. 0.7925, *P* = 0.002) and lower AIC (7178.448 vs. 7247.481) when compared to AS score system, which indicated integrating tumor grade, ER and PR status with AS could improve the stratification ability of score system. The estimated 4-years DSS outcomes for ILC cohorts categorized by AS+GEP score system were listed in Table 6. Sensitivity analyses conducted among patients without chemotherapy and patients with ER-positive and HER2-negative tumors also showed that the AS+GEP score system was superior to the AS score with lower AIC and higher C-index though the higher C-index was not statistically significant (Supplement Tables 3, 4).

**Table 6:** Survival outcomes by score group according to the AS+GEP score system.

**Score**	**No. of patients (%)**	**No. of DSS events**	**4-years DSS, %**	**95% CI, %**	**HR (95% CI)**	***P***	**No. of OS events**	**4-years OS**	**95% CI**	**HR (95% CI)**	***P***
0	2,416 (13.9)	6	99.41	98.64–99.75	1		51	96.65	95.46–97.54	1	
1	5,988 (32.4)	35	99.07	98.61–99.38	2.42 (1.02–5.75)	0.045	169	95.49	94.65–96.21	1.37 (1.00–1.88)	0.047
2	4,197 (22.5)	57	97.77	96.99–98.35	5.69 (2.45–13.20)	<0.001	172	93.43	92.24–94.45	2.02 (1.48–2.76)	<0.001
3	2,778 (15.1)	65	96.27	95.00–97.22	9.55 (4.14–22.04)	<0.001	146	91.90	90.31–93.24	2.53 (1.84–3.47)	<0.001
4	1,868 (10.1)	101	91.33	89.24–93.02	21.98 (9.65–50.09)	<0.001	163	86.59	84.22–88.63	4.17 (3.04–5.71)	<0.001
5	964 (5.2)	109	80.98	77.05–84.30	47.96 (21.08–109.09)	<0.001	149	75.33	71.22–78.95	7.68 (5.59–10.56)	<0.001
6	190 (1.0)	39	69.53	59.66–77.44	87.12 (36.88–205.79)	<0.001	43	66.79	56.93–74.89	11.27 (7.51–16.91)	<0.001
7	56 (0.3)	20	46.20	27.87–62.71	218.64 (87.79–544.53)	<0.001	22	43.99	26.50–60.18	27.73 (16.82–45.73)	<0.001

*AS+GEP, anatomic stage plus tumor grade plus estrogen receptor status and progesterone receptor status; CI, confidence interval; DSS, disease-specific survival; HR, hazard ratio; OS, overall survival*.

## Discussion

With the development of tumor biology research, it is well-acknowledged that biomarkers can provide additional prognostic information beyond tumor size and lymph node status (21–24). Accordingly, the AJCC 8th edition staging manual introduced ER, PR, HER2 status, and tumor grade into the staging system to refine risk stratification. Our study was conducted to validate and evaluate the pathological prognostic staging system in patients with IDC and ILC, two most common histology types in invasive breast cancer.

Previous studies have validated the superiority of the PS compared with the AS in predicting survival (8–11). Weiss et al. reported that the PS provided more accurate stratification compared with the AS in both cohorts from MD Anderson Cancer Center and from California Cancer Registry (10). Wang et al. verified that the PS improved the classification of patients with locally advanced breast cancer (11). Wong et al. further proved the superior ability of PS in predicting prognosis among Asian population (8). However, previous series mainly focused on the comparison of the AS and the PS in invasive breast cancer population dominated by IDC, while none compared them in different histological subtypes. The potential impact of histological subtypes on predictive value of the new staging system remained to be investigated.

To our knowledge, this study is the first large population-based report that validated the prognostic value of the PS from AJCC 8th edition staging manual in both IDC and ILC cohort. As described in previous series (14–16), distinctive differences in tumor features, treatment options, and recurrence patterns were observed between patients with IDC and patients with ILC. Therefore, it was of important significance to analyze the prognostic value of PS in the two different histological subtypes separately. In concordance with previously published studies (9, 25), the PS was superior to the AS in providing risk stratification information among patients with IDC. However, the PS didn't outperform the AS in predicting prognosis among patients with ILC according to our analyses.

The possible reasons may go as follows. To begin with, disparity between IDC and ILC in the distribution of clinicopathological features may have contributed to the divergent predictive performances of the AS and the PS. In line with previous series (14), our data showed that ILC was associated with heavier tumor burden at diagnosis, lower tumor grade, higher percentage of hormone receptor (HR)-positive, and HER2-negative tumors compared to IDC. Additionally, different prognostic importance of biomarkers weighed in IDC and ILC may influence the predictive performances of the PS. It has been reported that tumor grade similarly affected the prognosis of ILC and IDC but ER status and PR status were more important predictors for ILC (16). HER2 status was considered as strong prognostic factor in IDC, while in the current study, it failed to show correlation with DSS in ILC cohort. Furthermore, the premise of the utility of the PS was that patients have received appropriate regimens targeting the underlying biology of their breast cancers. ILC was reported to have better response to adjuvant endocrine therapy with survival improvements compared to matched IDC and (26, 27). Magnitude of benefit of adjuvant letrozole was also proved to be greater in ILC according to the analyses conducted in The Breast International Group (BIG) 1–98 population (27). The different response to systemic therapy between ILC and IDC may to some extent affect the efficacy of the PS in stratifying patients.

As the PS proposed in AJCC 8th edition staging manual failed to show superiority to AS in risk stratification among ILC patients, this study established a new risk-score point-based system specialized for ILC tumors to provide refinements on staging system. The major finding was that AS +GEP score system consisting of the AS, tumor grade, ER status, and PR status had the highest C-index and lowest AIC, indicating that the score system including biomarkers allowed for more refined patient classification in ILC population compared with that merely based on anatomic factors. According to AS+GEP score system, 68.8% of patients had a score of 0–2, with corresponding 4-years DSS > 97%. Our analyses indicated that the PS couldn't improve the risk stratification beyond AS after downstaging 50.5% of patients and upstaging 0.6% of patients. Different from PS, HER2 status was left out in the novel score system for it was not significantly associated with DSS according to our analyses, and this might lead to more rational stage migration. Moreover, the AS+GEP score system was more concise and easier to be used in clinical practice compared to PS. Sensitivity analyses conducted among patients without chemotherapy and patients with ER-positive and HER2-negative tumors further confirmed the superiority of AS+GEP score system with lower AIC and higher C-index compared to the AS score system though the higher C-index were not statistically significant. Because the AS+GEP score system incorporated ER status into the scoring system, its ability of risk stratification might be slightly weakened when analyses were restricted to ER-positive and HER2-negative patients. And the non-significant higher C-index of AS+GEP score system compared to AS among patients without chemotherapy might suggest that the superior predictive performance of AS+GEP score system was possibly due to its better risk stratification for patients with higher risk.

Limitations of the current study presented in the following aspects. One limitation lied in the lack of Oncotype DX recurrence score (RS) data in the present study. The PS incorporated RS into staging system and downstaged patients with T1-2N0M0, ER-positive, and HER2-negative tumors into stage IA when RS < 11 for the reason that these patients were observed exceptional survival outcomes (7). Similar with other published studies concerning the validation of PS, our analyses didn't include RS in PS due to the unavailability of RS data. Another limitation was that patients receiving neoadjuvant systemic therapy were unable to be excluded from the study cohort because of the insufficient treatment information provided by SEER database. However, subgroup analyses excluded patients with receipt of chemotherapy were conducted to alleviate bias, and similar results were observed which further confirmed our main finding. The relatively short follow-up was also a major limitation in our study. For the reason that HER2 status was not recorded in SEER database until 2010, patient selection was restricted to 2010–2015, which result in the limited follow-up. In particular, ILC was characterized by higher likelihood of late recurrence compared to IDC, so the median follow-up of 30.0 months might be inadequate for survival analyses in ILC cohort. Further studies with longer follow-up were needed to reach more robust conclusions. Moreover, information about the receipt of anti-HER2 therapy among HER2-poisitive patients was not provided in SEER database, which constituted another limitation of our study. However, a great majority of patients with HER2+ tumors may have received anti-HER2 therapy because only patients treated between 2010 and 2015 were included in the analyses. Other limitations consisted in those inherent in retrospective analyses.

## Conclusion

In conclusion, the current study validated that the PS was superior to AS in risk stratification among patients with IDC, while it failed to outperform AS among patients with ILC. Among risk score systems specially designed for ILC tumors, the AS+GEP score system could provide more precise prognostic information. Further studies should strive to refine staging system for patients with specific breast cancer subtypes.

## Data Availability Statement

Publicly available datasets were analyzed in this study. This data can be found here: National Cancer Institute's SEER program (https://seer.cancer.gov/).

## Author Contributions

SD, JW, and LZ: conceptualization. CL, WeilC, and DL: methodology. SD and JW: formal analysis, investigation, and writing–original draft preparation. LZ and YZ: writing–review and editing. LZ: funding acquisition. WeigC, YL, KS, and LZ: supervision. All authors contributed to the article and approved the submitted version.

## Conflict of Interest

The authors declare that the research was conducted in the absence of any commercial or financial relationships that could be construed as a potential conflict of interest.
