# Supplementary material for: Validation of the Prognostic Stage of American Joint Committee on Cancer Eighth Edition Staging Manual in Invasive Lobular Carcinoma Compared to Invasive Ductal Carcinoma and Proposal of a Novel Score System
Source: Front Oncol. 2020 Aug 18;10:1471. doi: 10.3389/fonc.2020.01471 (PMC7461987; doi:10.3389/fonc.2020.01471)
Supplement: Supplementary file 1 [file Data_Sheet_1.docx]

Supplement Table1. Comparisons of different staging systems after excluding patients with chemotherapy.

| Endpoint | Staging system | IDC cohort | | | ILC cohort | | |
| --- | --- | --- | --- | --- | --- | --- | --- |
|  |  | AIC | C-index | P for  C-index | AIC | C-index | P for  C-index |
| DSS |  |  |  |  |  |  |  |
|  | AS | 34505.23 | 0.8625 | <0.001 | 3409.928 | 0.8713 | 0.090 |
|  | PS | 33789.74 | 0.8802 |  | 3423.048 | 0.8591 |  |
| OS |  |  |  |  |  |  |  |
|  | AS | 98472.24 | 0.8007 | 0.003 | 10475.35 | 0.7901 | 0.027 |
|  | PS | 97916.33 | 0.8052 |  | 10493.56 | 0.7803 |  |

Abbreviations: IDC, invasive ductal carcinoma; ILC, invasive lobular carcinoma; DSS, disease-specific survival; OS, overall survival; CI, confidence interval; AS, anatomic staging system; PS, prognostic staging system; AIC, Akaike information criterion; C-index, concordance index

Supplement Table2. Comparisons of different staging systems among patients with ER-positve and HER2-negative tumors

| Endpoint | Staging system | IDC cohort | | | ILC cohort | | |
| --- | --- | --- | --- | --- | --- | --- | --- |
|  |  | AIC | C-index | P for  C-index | AIC | C-index | P for  C-index |
| DSS |  |  |  |  |  |  |  |
|  | AS | 44883.96 | 0.8219 | P<0.001 | 6019.248 | 0.8345 | P=0.011 |
|  | PS | 44288.19 | 0.8370 |  | 6050.292 | 0.8169 |  |
| OS |  |  |  |  |  |  |  |
|  | AS | 103387.6 | 0.7667 | P<0.001 | 14002.89 | 0.7789 | P=0.070 |
|  | PS | 102966 | 0.7746 |  | 14021.42 | 0.7730 |  |

Abbreviations: IDC, invasive ductal carcinoma; ILC, invasive lobular carcinoma; DSS, disease-specific survival; OS, overall survival; CI, confidence interval; AS, anatomic staging system; PS, prognostic staging system; AIC, Akaike information criterion; C-index, concordance index

Supplement table 3 Comparisons of different scoring systems after excluding patients with chemotherapy(n=12552).

| Scoring system | AIC | C-index | P for C-index |
| --- | --- | --- | --- |
| AS | 3468.831 | 0.8214 | / |
| AS+G | 3464.273 | 0.8198 | 0.109 |
| AS+GEP | 3449.534 | 0.8302 | 0.262 |

Abbreviations: AS, anatomic stage; AS+G, anatomic stage plus grade; AS+GEP, anatomic stage plus grade plus estrogen receptor plus progesterone receptor; AIC, Akaike information criterion; C-index, concordance index

Supplement table 4 Comparisons of different scoring systems among patients with ER-positive and HER2-negative tumors(n=17381).

| Scoring system | AIC | C-index | P for C-index |
| --- | --- | --- | --- |
| AS | 6134.929 | 0.8362 | / |
| AS+G | 6134.638 | 0.8310 | 0.152 |
| AS+GEP | 6116.453 | 0.8356 | 0.456 |

Abbreviations: AS, anatomic stage; AS+G, anatomic stage plus grade; AS+GEP, anatomic stage plus grade plus estrogen receptor plus progesterone receptor; AIC, Akaike information criterion; C-index, concordance index
